# Comparison of Pelvic Organ Prolapse and Sexual Function After Vaginal Natural Orifice Transluminal Endoscopic Surgery and Conventional Laparoscopic Hysterectomy

**DOI:** 10.3390/jcm14082590

**Published:** 2025-04-09

**Authors:** Mesut Ali Halisçelik, Cengiz Şanlı, Mesut Bala, Selami Erdem, Behzat Can, İhsan Bağlı, Mehmet Obut, Sedat Akgöl, Cihan Bademkıran, Kevser Arkan, Ali Deniz Erkmen, Serhat Ege, Süleyman Cemil Oğlak, Salih Burçin Kavak

**Affiliations:** 1 Department of Obstetrics and Gynecology, Diyarbakir Gazi Yasargil Research and Training Hospital, University of Health Sciences, 21070 Diyarbakır, Turkey; mesuthaliscelik@hotmail.com (M.A.H.); mesut.bala@gmail.com (M.B.); drbehzatcan@gmail.com (B.C.); ihsanbagli@gmail.com (İ.B.); drmehmetobut@hotmail.com (M.O.); drsedatakgol@hotmail.com (S.A.); cihan352135@gmail.com (C.B.); kevser.toprak1989@gmail.com (K.A.); erkmendnz@hotmail.com (A.D.E.); drcemiloglak@gmail.com (S.C.O.); 2 Department of Obstetrics and Gynecology, Fethi Sekin City Hospital, University of Health Sciences, 23119 Elazığ, Turkey; 3 Department of Obstetrics and Gynecology, Private Batı Hospital, 21070 Diyarbakır, Turkey; serdem2109@gmail.com; 4 Department of Obstetrics and Gynecology, Dicle University, 21070 Diyarbakır, Turkey; serhatege782@gmail.com; 5 Department of Obstetrics and Gynecology, Fırat University, 23119 Elazığ, Turkey; burcinkavak1@gmail.com

**Keywords:** hysterectomy, laparoscopy, female sexual function index, pelvic organ prolapse

## Abstract

**Aim**: In our study, we aimed to comprehensively evaluate the surgical parameters of patients who underwent total laparoscopic hysterectomy (TLH) and vaginal natural orifice transluminal endoscopic surgery (vNOTES) hysterectomy and their effects on the development of pelvic organ prolapse (POP) and sexual function one year later. **Materials and Methods**: This retrospective and comparative study involved a review of medical records for 42 patients who underwent total laparoscopic hysterectomy (TLH) for benign conditions and 42 patients who underwent hysterectomy using vaginal natural orifice transluminal endoscopic surgery (vNOTES) at our clinic between January 2023 and January 2024. Evaluations of preoperative and postoperative pelvic organ prolapse and sexual function were conducted, and the anatomical and functional outcomes of both surgical methods were compared. **Results**: In this study, there was no statistically significant difference between the groups in terms of age (*p* < 0.299), BMI (*p* < 0.819), parity (*p* < 0.615), surgical indications and menopausal status (*p* < 0.535) (*p* > 0.05). The vNOTES method was found to be significantly shorter than TLH in terms of surgical time (vNOTES: 58 min, TLH: 80 min, *p* < 0.001). However, there was no significant difference between the two methods in terms of preoperative and postoperative hematocrit values, hospital stay and FSFI scores (*p* > 0.05). Among the POP-Q parameters, a significant difference was observed only in the Aa parameter in favor of the vNOTES method (*p* < 0.003). **Conclusions**: In our study, the vNOTES method provided a shorter surgical time and better anterior vaginal support compared to TLH, but both methods offered similar results in terms of sexual function. More comprehensive studies are needed to clarify the long-term effects.

## 1. Introduction

Hysterectomy is the most frequently performed gynecological surgery globally, with approaches including endoscopic, abdominal and vaginal methods [1]. Vaginal hysterectomy (VH) is the preferred surgical technique for suitable patients [2], as it is less invasive compared to other methods and offers benefits such as reduced recovery time, lower risk of infection, and minimal postoperative pain. However, its application in clinical practice may be constrained by limited imaging capabilities and challenges in manipulation techniques [3]. Abdominal hysterectomy (AH) is associated with an increased risk of pelvic organ prolapse (POP) in comparison to other surgical procedures, as it results in greater damage to pelvic support tissues [4,5]. With the advent and widespread adoption of endoscopic surgery, there has been a noted decline in the rates of abdominal hysterectomy, while the incidence of laparoscopic hysterectomy has progressively risen [6]. Laparoscopic hysterectomy is favored over open abdominal hysterectomy for patients for whom vaginal hysterectomy is not feasible [7]. The NOTES technique, which involves accessing the peritoneal cavity through natural orifices, was first introduced in 2004 [8]. A study conducted in 2012 established that the NOTES technique, when performed vaginally (vNOTES), is both safe and feasible. This research indicated that surgeries conducted through natural orifices yield successful outcomes when executed by experienced surgeons and with appropriate patient selection [9]. VNOTES represents an innovative surgical approach that has gained increasing attention in recent years. This technique integrates the minimally invasive characteristics of endoscopic procedures with the benefits of vaginal surgery, utilizing natural orifices [10]. Studies have demonstrated that the vNOTES technique offers several advantages over conventional laparoscopy. It has been reported that this method results in reduced intraoperative blood loss, decreased postoperative pain, and an accelerated recovery process. Furthermore, vNOTES is aesthetically advantageous, as it leaves no abdominal incision scar, thereby providing superior cosmetic outcomes [11,12,13].

Pelvic organ prolapse (POP) is a prevalent health issue that substantially impacts women’s daily activities, social interactions, and sexual functioning [14]. Pelvic organ prolapse (POP) is characterized by the downward displacement of one or more pelvic organs, including the anterior vaginal wall, posterior vaginal wall, uterus, or vaginal cuff. This condition may present in patients with a variety of symptoms, such as urinary and defecation issues, pelvic pain, and sexual dysfunction [7]. Hysterectomy can impact the anatomical and neurological integrity of the pelvic floor, potentially leading to its weakening and thereby increasing the risk of POP. However, in addition to hysterectomy, factors such as age, obesity, vaginal delivery, parity, and the underlying reason for surgery also significantly contribute to the development of POP [4,5,15].

Hysterectomy may lead to pelvic floor weakness by modifying anatomical structures and nerve supply [4,5,15]. The incidence of vaginal cuff prolapse necessitating surgical intervention following hysterectomy exhibits variability across different studies in the literature. One study reported this rate to be 6.25% [16], whereas another study found the incidence of prolapse requiring surgical correction post-hysterectomy to range between 1.3 and 4.2 per 1000 woman-years [17]. These findings indicate that the risk of vaginal cuff prolapse after hysterectomy should be regarded as a significant clinical concern. Vaginally performed vNOTES results in reduced trauma to the abdominal muscles and pelvic anatomy, as it is conducted without breaching the anterior abdominal wall. Consequently, this approach may lead to a decreased incidence of prolapse and sexual dysfunction. This study aimed to conduct a comparative evaluation of the surgical parameters, the development of pelvic organ prolapse (POP) during a one-year follow-up period and the effects on sexual function in patients who underwent total laparoscopic hysterectomy (TLH) and vNOTES hysterectomy.

## 2. Materials and Methods

In this retrospective, comparative study, the medical records of patients who underwent total laparoscopic hysterectomy (TLH) (group 1) and vaginal natural orifice transluminal endoscopic surgery (vNOTES) (group 2) hysterectomy between January 2023 and January 2024 at the Gynecology and Obstetrics Clinic of the University of Health Sciences Gazi Yasargil Training and Research Hospital were reviewed. The study adhered to the ethical principles for medical research involving human subjects as outlined in the Declaration of Helsinki (Diyarbakır Gazi Yaşargil Training and Research Hospital Ethics Committee no: 298/2024).

The study cohort comprised 84 patients, aged 30 to 80 years, who underwent 42 vNOTES hysterectomies and 42 TLHs for benign uterine pathologies. The primary objective was to compare the impact of these two hysterectomy techniques on pelvic organ prolapse and female sexual function in the postoperative period.

Inclusion criteria encompassed patients who underwent hysterectomy for benign conditions such as uterine leiomyomas (fibroids), adenomyosis, endometrial hyperplasia, abnormal uterine bleeding, cervical dysplasia, or cervical intraepithelial neoplasia, who had stage 0 or 1 pelvic organ prolapse according to the pelvic organ prolapse quantification (POP-Q) system prior to surgery, who were sexually active, and who were followed up for a minimum of 12 months post-surgery. Exclusion criteria included patients who underwent hysterectomy for malignant pathologies, those with a diagnosis of pelvic organ prolapse of stage 2 or higher according to the POP-Q system, those with a history of previous pelvic surgery, intraoperative complications, chronic pelvic pain, neurological diseases, or pelvic radiation history, and those lacking complete follow-up data within 12 months post-surgery.

During the postoperative follow-up period, patient records were retrospectively analyzed 12 months after the operation. This analysis utilized the pelvic organ prolapse quantification (POP-Q) system for evaluating pelvic organ prolapse and the Female Sexual Function Index (FSFI) scale for assessing sexual function. The FSFI, which was developed to evaluate female sexual function and possesses established scientific validity, is widely employed as a reliable assessment tool. The FSFI aims to objectively measure female sexual function and is extensively used in clinical applications and research. The FSFI evaluates women’s sexual function across domains such as sexual desire, arousal, vaginal lubrication, orgasm, sexual pain, and satisfaction, with each domain scored from 0 to 6, providing a comprehensive assessment of women’s sexual health status. FSFI questionnaires were administered to patients both pre- and post-surgery.

The pelvic organ prolapse quantification (POP-Q) system is a widely utilized method for grading pelvic organ prolapse and assessing pelvic floor function in women. This system determines the severity of prolapse through precise measurements taken at various anatomical landmarks [14]. Measurements were conducted during preoperative and postoperative follow-up appointments, with patients positioned in the lithotomy position and performing the Valsalva maneuver to evaluate the severity of pelvic organ prolapse. Total vaginal length measurements were recorded while the patient was at rest. During these evaluations, measurements in centimeters were taken at two points in the anterior compartment (Aa and Ba), two points in the posterior compartment (Ap and Bp), the genital hiatus (gh), the perineal body (pb) and the vaginal length.

The staging of pelvic organ prolapse was based on the most distal point of prolapse, using the hymen as the reference point. This retrospective study facilitated a comprehensive evaluation of anatomical and functional outcomes following surgery. All patients underwent a preoperative gynecologic examination and cervical smear test and were assessed via ultrasonography. Informed consent was obtained from all patients preoperatively. Data on age, body mass index (BMI), parity, age, and surgical indications were recorded. Additionally, preoperative and postoperative hematocrit values, operation duration, and hospitalization time were documented. All surgical procedures were performed by surgeons with advanced expertise in gynecologic endoscopic surgery.

Both groups underwent surgical procedures under general anesthesia, positioned in the lithotomy stance with endotracheal intubation. Preoperatively, patients received 2 or 3 g of intravenous cefazolin, contingent upon their body mass index (BMI). During the operation, the insertion of urinary and nasogastric catheters was implemented for all patients, a standard practice aimed at ensuring the safety and efficiency of the surgical process.

### 2.1. vNOTES Hysterectomy Procedure

Patients were positioned in the high lithotomy position. The anterior and posterior cervical lips were secured using a tenaculum. A circumferential cervical incision was executed utilizing monopolar cautery. The cervical fascia was meticulously separated from the vaginal mucosa through dissection. A posterior colpotomy was created via sharp dissection. Subsequently, the cardinal and sacrouterine ligaments were grasped, transected, ligated, and suspended with sutures. An anterior colpotomy was then conducted. The abdominal cavity was accessed with a retractor, and the abdomen was inspected. Following this, the metal retractors were removed, and the GelPoint vPath (Applied Medical, Rancho Santo Margarita, CA, USA) was inserted. Pneumoperitoneum was established using CO_2_ insufflation, with the pressure adjusted to 12 mmHg. A total of three trocars were employed. A 30° camera was introduced through a 10 mm trocar, while additional laparoscopic instruments were inserted through 5 mm trocars. Bipolar electrocoagulation (LigaSure™, Covidien Company, Mansfield, MA, USA) was utilized for tissue dissection. The uterine vessels, adnexal pedicles, and infundibulopelvic ligaments were transected in both caudal and cranial directions. The specimen was extracted vaginally. The vaginal cuff was peritonized and sutured using 1-0 (Vicryl^®^, Ethicon, Piscataway, NJ, USA) sutures.

### 2.2. TLH Procedure

All patients underwent surgery in the lithotomy position. A Veress needle (Ethicon Endo-surgery, Inc., Piscataway, NJ, USA) was introduced through the umbilical incision, and the abdomen was insufflated with CO_2_ to a pressure of 20 mmHg. A 10 mm trocar was inserted into the abdomen via the umbilicus, followed by the insertion of a 30° 10 mm optical trocar. The patient was then positioned in the Trendelenburg position. Subsequently, the pressure was reduced to 15 mmHg. A uterine manipulator (RUMI-II^®^, CooperSurgical, Trumbull, CT, USA) was employed to manipulate the uterus in all cases. Upon visualization of all abdominal organs, 5 mm trocars were inserted, two on the left and one on the right. Bipolar electrocoagulation (LigaSure™, Covidien Company, Mansfield, MA, USA) was utilized as the energy modality. The uterine artery and ligaments were transected in a cranial to caudal direction. The bladder was dissected away from the uterus. The parametrial tissue surrounding the cervix was dissected with the assistance of LigaSure. A circular colpotomy was performed over the uterosacral ligament using monopolar cautery, and the uterus was completely detached from the vagina. The uterus and adnexa were extracted from the abdomen through the vagina. The vaginal cuff was closed using laparoscopic 1-0 suture (Vicryl^®^, Ethicon, Piscataway, NJ, USA).

### 2.3. Power Analysis

A post hoc power analysis was conducted for the Mann–Whitney U test employed in this study. Assuming a moderate effect size (Cohen’s d = 0.55), with a total sample size (n = 84) and an error rate of 5% (α = 0.05), the statistical power of the study was calculated to be 80.4%. The 80% power level, which is generally accepted in the literature, suggests that the probability of detecting a significant effect is adequate. Therefore, the obtained value of 80.4% is deemed appropriate in terms of the study’s reliability.

### 2.4. Statistical Analysis Method

In this study, SPSS 26 software was employed for all statistical analyses. For the comparison of the TLH and vNOTES groups across various parameters, descriptive statistics, including median, minimum, maximum, frequency, and percentage values, as well as central tendencies and distributions of variables, were summarized in accordance with the data characteristics. The Mann–Whitney U Test, which is appropriate for evaluating differences between two independent groups when parametric assumptions are unmet, was utilized. Additionally, the Chi-Square (χ^2^) Test was applied to compare categorical variables. Furthermore, the Wilcoxon Signed Rank Test was employed for comparisons between two dependent groups to assess differences between preoperative and postoperative measurements. This test was specifically chosen to evaluate changes in preoperative and postoperative variables for both surgical methods. Notably, Wilcoxon analysis was conducted for data such as vaginal length, preoperative and postoperative pelvic anatomical variables (Aa, Ap, Ba, Bp, Gh), and hematocrit levels, with statistically significant changes observed postoperatively.

## 3. Results

When the demographic and clinical characteristics of the patients in both groups were analyzed in Table 1, it was observed that the majority of the patients were in menopause and their body mass index (BMI) values were below 30. In terms of age distribution, the median age was below 55 years in both groups. The median age was 49 (32–73) in the TLH group and 51 (35–75) in the vNOTES group. BMI values were similar in both groups with a median of 28 (21–40). When menopausal status was evaluated, 65.1% (n = 28) of the TLH group and 58.5% (n = 24) of the vNOTES group were in menopause. There was no statistical difference between the groups in terms of age (*p* < 0.299), BMI (*p* < 0.819), parity (*p* < 0.615), surgical indications and menopausal status (*p* < 0.535) (*p* > 0.05). These findings revealed that both groups had similar characteristics in terms of age, BMI, menopausal status and indications. Statistical analysis showed that there were no differences between the groups in terms of age (*p* = 0.299), BMI (*p* = 0.819), menopausal status (*p* = 0.535) and indications (*p* = 0.362). The most common indications for surgery in patients undergoing vNOTES and TLH were myoma uteri and abnormal uterine bleeding. Myoma uteri was detected in 53.7% (n = 22) of patients in the vNOTES group and 44.2% (n = 19) in the TLH group, while abnormal uterine bleeding was observed in 29.3% (n = 12) in the vNOTES group and 25.6% (n = 11) in the TLH group. There was no significant difference between TLH and vNOTES groups in terms of surgical indications (*p* > 0.05).

These findings suggest that TLH and vNOTES methods can be safely applied in patient groups with similar demographic and clinical characteristics.

Table 2 provides a comparative analysis of the TLH and VNOTES techniques with respect to surgical duration, as well as preoperative and postoperative hematocrit levels and hospitalization duration. The VNOTES group demonstrated a significantly reduced surgical time (58) compared to the TLH group (80) (*p* < 0.001). However, no statistically significant differences were observed between the two methods concerning preoperative and postoperative hematocrit values or the length of hospitalization (*p* > 0.05).

Table 3 presents a comparison of preoperative and postoperative parameters for the TLH and vNOTES methods, specifically focusing on POP-Q (pelvic organ prolapse quantification) and FSFI (Female Sexual Function Index) scores. The analysis revealed no significant difference in preoperative FSFI values between the TLH and vNOTES groups (*p* > 0.05), suggesting comparability in terms of FSFI. Similarly, the comparison of postoperative FSFI values indicated no significant difference between the two groups (*p* > 0.05).

In this study, a comparison of the preoperative POP-Q parameters between the groups revealed that all parameters were similar, with the exception of Aa (*p*). Postoperative analysis indicated that the v-NOTES group exhibited superior outcomes in terms of POP-Q parameters Aa, Ba, gh, and vaginal length. However, when evaluating the impact of surgery on these parameters through preoperative and postoperative change analysis, the v-NOTES group demonstrated a statistically significant improvement over the TLH group only in the Aa parameter (*p* < 0.003), while the gh variable approached borderline significance with *p* = 0.051. Furthermore, the definitive pathological findings in patients with a preliminary diagnosis of abnormal uterine bleeding were consistent with preoperative assessments, including proliferative or secretory endometrium, atypical endometrial hyperplasia, cervical carcinoma in situ (notably in two young patients in their thirties), and endometrial intraepithelial neoplasia.

The definitive pathological findings for patients initially diagnosed with abnormal uterine bleeding were consistent with conditions such as proliferative or secretory endometrium, atypical endometrial hyperplasia, endometrial intraepithelial neoplasia, and cervical carcinoma in situ. Notably, these diagnoses were observed in two young patients in their thirties. Additionally, multiple and widely distributed fibroids were identified in both patient cohorts. Pathological examination of all myoma cases confirmed benign characteristics.

## 4. Discussion

Hysterectomy is among the most frequently performed procedures in gynecologic surgery; however, it is associated with various complications in both the early and late postoperative periods. Notably, pelvic organ prolapse (POP) and sexual dysfunction are significant concerns [1,15]. This study aims to compare the incidence of POP and the impact on sexual function in patients undergoing hysterectomy via total laparoscopic hysterectomy (TLH) and vaginal natural orifice transluminal endoscopic surgery (vNOTES) methods.

Hysterectomy may pose a risk factor for POP by compromising the anatomical and neurological integrity of the pelvic floor. Several studies indicate that vaginal support mechanisms may be weakened post-hysterectomy, thereby increasing the risk of POP [18]. In the context of benign indications, no significant difference in POP prevalence was observed between TLH and vaginal hysterectomy (VH) in non-prolapse conditions [18]. Aigmueller et al. reported a 6.25% incidence of prolapse necessitating surgery following hysterectomy [16], whereas Dallenbach et al. documented a need for surgery at a rate of 1.3–4.2 per 1000 per year [17]. In the present study, no patients required surgical intervention for postoperative prolapse after a one-year follow-up. This discrepancy may be attributed to the relatively short duration of the follow-up period.

In the preoperative evaluation of patients in both groups, the stage was classified as 0–1 according to the pelvic organ prolapse quantification (Pop-Q) system. Postoperatively, patients in both groups were similarly assessed as stage 0–1 using the Pop-Q staging. A notable strength of this study is the absence of preoperative prolapse in both groups, which underscores the role of hysterectomy in the etiology of prolapse. However, the vNOTES approach yielded superior anatomical outcomes in postoperative Ba (*p* = 0.015), postoperative Aa (*p* = 0.011), and postoperative Gh (*p* = 0.044). These findings indicate that vNOTES may offer enhanced improvement in anterior vaginal wall support and potentially augment pelvic support in this region.

It is well established that factors such as age, obesity, a history of vaginal delivery and connective tissue weakness significantly contribute to the development of POP [19]. The incidence of pelvic organ prolapse (POP) is significantly correlated with increasing age [20]. Childbirth is identified as the primary risk factor for POP. Current evidence indicates that the process of childbirth results in damage to the nerves, fascia and muscles of the pelvic floor [21]. Obesity constitutes a significant risk factor for pelvic floor dysfunction. The primary cause of pelvic organ prolapse (POP) in obese women is increased intra-abdominal pressure, which leads to the weakening of pelvic floor tissues [22]. This study’s findings reveal that the demographic and clinical characteristics of both surgical groups were similar, suggesting that the observed outcomes are attributable to differences in surgical intervention, thereby enhancing the study’s comparability.

Pelvic organ prolapse (POP) is identified during physical examinations in approximately 41% to 50% of women, yet only 3% exhibit symptoms [23]. In this study, both symptomatic and asymptomatic patients were assessed preoperatively and postoperatively to more accurately determine the true incidence of pelvic organ prolapse. This methodological rigor significantly contributes to the scientific validity of the study’s findings.

In our study, we found that vNOTES (58) was statistically significantly shorter than TLH (80) in terms of surgical time (*p* < 0.001). The use of vNOTES may be attributed to its facilitation of surgical access through natural orifices, thereby minimizing the need for additional trocar placement and reducing the dissection times typically required during laparoscopic procedures. Furthermore, previous studies have demonstrated that vNOTES is associated with shorter surgical durations and enhances the efficiency of the operative process [12].

Female sexual dysfunction is a complex condition influenced by the interplay of physiological, psychological, and social factors. The impact of hysterectomy on sexual function varies [24]. Although it is commonly believed that hysterectomy does not adversely affect sexual function, sexual issues may arise due to factors such as vaginal narrowing, nerve damage, and psychological influences [25,26]. The effects of different types of hysterectomy on sexual function have not been fully elucidated. In a study comparing total abdominal hysterectomy and vaginal hysterectomy (VH), it was observed that vaginal length decreased and the incidence of dyspareunia increased in patients who underwent vaginal hysterectomy [27]. Another study showed significant improvements in sexual function and quality of life in the postoperative period, regardless of the type of hysterectomy performed [24]. In our study, the sexual functions of the patients were evaluated with FSFI scores and no significant change was observed in the preoperative and postoperative periods for either surgical method (*p* > 0.05). Although a change in vaginal length was observed following hysterectomy, this difference did not reach statistical significance (*p* = 0.340). The findings clearly indicate that neither surgical method significantly impacts sexual function or vaginal length. These results imply that the short-term effects of surgery are limited, necessitating more comprehensive and long-term studies to ascertain the long-term effects. Furthermore, the results suggest that vNOTES yields outcomes comparable to TLH in terms of sexual function, while offering superior anatomical results. A notable strength of this study is its documentation of preoperative and postoperative FSFI values, allowing for the observation of these changes. Consequently, it was concluded that the impact of hysterectomy on sexual function was not statistically significant.

In patients presenting with an enlarged uterus and multiple fibroids, vNOTES is a beneficial technique as it facilitates access to uterine vessels and permits the safe dissection of ligaments under direct optical visualization [28]. In our study, multi-tip fibroids were detected in 19 (44.2%) of the patients in the vNOTES group, and all these cases were treated successfully and without complications without the need for an additional surgical method.

Housmans et al. reported that vNOTES resulted in shorter operative time, shorter hospital stay and lower estimated blood loss [24]. In our study, a similar statistically significant difference was observed between the groups regarding the duration of surgery (VNOTES:58 TLH:80 *p* < 0.001). However, we did not find any significant difference between preoperative and postoperative hematocrit values and length of hospitalization. Compared to TLH, VNOTES offers significant advantages such as shorter surgical time and faster postoperative recovery, in addition to allowing clearer visualization of pelvic anatomical structures [29]. Factors such as cosmetic advantages due to the absence of scar formation and decreased risk of trocar-related complications have been effective in increasing the prevalence of this technique [10]. In the present study, it was observed that patients reported significant satisfaction with the cosmetic outcomes at the one-year postoperative follow-up. These findings suggest that vNOTES may offer a more advantageous alternative to TLH. Moreover, further research is warranted to assess the impact of hysterectomy on the development of prolapse and the long-term effects of the surgical technique on pelvic floor integrity. However, the study is subject to certain limitations, including its retrospective design, relatively small patient cohort and the absence of long-term follow-up data. It is recommended that future large-scale, randomized controlled trials be conducted to evaluate the long-term outcomes of vNOTES in a more comprehensive manner.

## 5. Conclusions

The vNOTES technique represents a significant option in pelvic surgery practice, which is attributable to its reduced surgical duration, enhanced anterior vaginal support, and cosmetic benefits in comparison to TLH. Furthermore, the comparative assessment of the impacts of various surgical techniques on the anterior and posterior compartments of the vagina offers valuable clinical contributions to the literature. Nonetheless, further research is required to elucidate the long-term outcomes of this method and its implications for the development of POP.

## Figures and Tables

**Table 1:** Comparison of surgical approaches according to demographic and clinical characteristics.

Variables		TLH Median (Min–Max)	vNOTES Median (Min–Max)	U-X^2^	*p*
Age		49 (32–73)	51 (35–75)	765,500	0.299
BMI		28 (21–40)	28 (21–40)	856,500	0.819
Parity		4 (0–7)	4 (0–12)	826,000	0.615
Indications	Abnormal Uterine Bleeding	11 (25.6)	12 (29.3)	4341	0.362
	Uterine Myoma	19 (44.2)	22 (53.7)		
	Endometrial Hyperplasia	9 (20.9)	4 (9.8)		
	Cervical Intraepithelial Lesion	2 (4.7)	3 (7.3)		
	Adenomyosis	2 (4.7)	0 (0.0)		
Menopause	Positive	28 (65.1)	24 (58.5)	0.385	0.535
	Negative	15 (34.9)	17 (41.5)		

BMI: body mass index; *p* < 0.05 was considered statistically significant.

**Table 2:** Comparison of surgical time, hematocrit values and length of hospitalization.

Variables	TLH Median (Min–Max)	vNOTES Median (Min–Max)	U	*p* *
Surgical Duration (minute)	80 (48–90)	58 (55–79)	103,000	0.001 *
Preoperative Htc	40 (30–48)	40 (29–47)	879,000	0.982
Postoperative Htc	34 (24–43)	35 (24–44)	869,000	0.911
Length of Stay	2 (1–5)	2 (1–4)	739,000	0.148

* *p* < 0.05 was considered statistically significant. Htc: hematocrit.

**Table 3:** TLH and vNOTES comparison of POP-Q and FSFI scores and change before and after surgery.

Variables	Tlh Median (Min–Max)	Vnotes Median (Min–Max)	U	*p*
Preoperative Op FSFI	26.3 (20.3–31)	26.5 (19–31.2)	874,500	0.950
Preoperative Op FSFI	24.4 (17.2–29.6)	24.4 (17.2–29.6)	880,500	0.993
**Preoperative POP-Q**				
Preoperative Op Aa	−2 (−3–−1)	−2 (−3–−1)	862,500	0.853
Preoperative Op Ba	−3 (−3–0)	−3 (−3–−2)	640,000	0.012
Preoperative Op Ap	−2 (−3–0)	−2 (−3–2)	834,500	0.645
Preoperative Op Bp	−3 (−3–−3)	−3 (−3–−3)	881,500	1.000
Preoperative Op Gh	5.3 (3.2–7.0)	5.5 (3.8–7.2)	603,000	0.166
Preoperative Op Pb	3 (1.8–4.6)	3 (2–4.4)	863,300	0.164
Preoperative TVL	8 (7–10)	8 (7–9)	804,000	0.445
**Postoperative POP-Q**				
Postoperative Op Aa	−2 (−3–0)	−2 (−3–−1)	623,500	0.011
Postoperative Op Ba	−2 (−3–1)	−3 (−3–−2)	640,000	0.015
Postoperative Op Ap	−2 (−3–0)	−2 (−3–2)	741,000	0.166
Postoperative Op Bp	−3 (−3–−2)	−3 (−3–−2)	807,500	0.369
Postoperative Vaginal Length	7 (6–9)	8 (6–9)	784,500	0.340
Postoperative Op Gh	4.5 (3–7)	5 (3.5–7)	658,500	0.044
Postoperative Op Pb	3 (1.8–4.4)	3 (1.9–4.4)	855,000	0.811
**Preoperative and postoperative POP-Q and FSFI change**				
FSFI	2 (0–4.1)	2 (0–3)	814,000	0.543
Aa	0 (−2–1)	0 (−1–2)	590,500	0.003
Ba	0 (−3–0)	0 (−1–0)	823,500	0.456
Ap	0 (−1–0)	0 (−1–0)	742,000	0.060
Bp	0 (−1–0)	0 (−1–0)	807,500	0.369
TVL CHANGE	1 (0–2)	1 (0–2)	837,000	0.605
Gh	0.2 (0–0.6)	0.3 (0–1)	666,000	0.051
Pb	1 (0–0.2)	0 (0–0.4)	860,500	0.837

*p* < 0.05 was considered statistically significant. Aa; anterior point A: Ba; anterior point B; Gh; genital hiatus: Pb; perineal body: Ap; posterior point A. Bp; posterior point B: TVL; total vaginal length. POP-Q; pelvic organ prolapse quantification. FSFI; female Sexual Function Index.

## Data Availability

Data are contained within the article.

## Abbreviations

The following abbreviations are used in this manuscript:

TLH	Total laparoscopic hysterectomy.
Vnotes	Vaginal natural orifice transluminal endoscopic surgery.
POP	Pelvic organ prolapse.
POPQ	Pelvic organ prolapse quantification.
FSFI	Female Sexual Function Index.
BMI	Body mass index.
TVL	Total vaginal length.
Aa and Ba	Anterior wall.
GH	Genital hiatus.
PB	Perineal body.
Ap and Bp	Posterior wall.
VH	Vaginal hysterectomy.
